# Importation and distribution of unregistered medicines in the public sector: similarities, differences, and shared challenges among Southern African Development Community (SADC) countries

**DOI:** 10.1186/s12913-022-07995-3

**Published:** 2022-04-28

**Authors:** Admire Nyika, Bernard Ngara, Isaac Mutingwende, Luther Gwaza

**Affiliations:** 1 Department of Pharmacy & Pharmaceutical Sciences, Faculty of Medicine and Health Sciences, University of Zimbabwe, Mazowe Street, Parirenyatwa Complex, P.O Box A178, Avondale, Harare Zimbabwe; 2 World Health Organization, Health Products Policy and Standards, Access to Medicines and Health Products Division, 20 Appia Avenue, Geneva, Switzerland

**Keywords:** Importation, Unregistered medicines, Legal provisions, Implementation, SADC

## Abstract

**Background:**

The purpose of the study was to assess the requirements for approval of the importation of unregistered medicines for use in the public sector in the Southern African Development Community (SADC) countries.

**Methods:**

The study reviewed the legal provisions and requirements to be fulfilled when importing unregistered medicines for the public sector in SADC countries relative to two comparators drawn from countries with stringent regulatory systems through extant document analysis. The relative implementation index score was calculated and used to measure the level of implementing legal provisions and requirements to be fulfilled. Analysis was performed using the STATA software package.

**Results:**

Approximately 13 out of 16 SADC countries had a relative implementation index below 50%. The aggregated implementation index across all SADC countries was 44%, ranging from 4 to 54%, while the two comparators had a relative implementation index of 81% and 85%, respectively.

**Conclusion:**

Implementing the minimum requirements for importing unregistered medicines for the public sector was deficient compared to the jurisdictions with stringent regulatory systems, and wide implementation gaps also existed within the SADC region.

## Background

The use of medical products such as pharmaceuticals, biologicals including vaccines, blood products, and medical devices, including in vitro diagnostics, is essential in the healthcare provision system [1, 2]. Every country should ensure an adequate supply of safe, efficacious, good quality, and affordable medical products to promote public health [3].Effective regulatory systems ensure that the medical products meet the recommended standards to protect and promote public health [4, 5]. All countries globally are encouraged to have functional, effective, and efficient National Regulatory Authorities (NRAs) [6]. However, at least 30% of the existing NRAs have constrained capacity to perform core regulatory functions [7, 8].

Medical products are allowed for use after the respective NRAs approve them. The requirements to obtain such approval differ among NRAs based on their regulatory capacity [9, 10]. The regulatory review process requires a considerable amount of time and adequate skilled personnel, impacting the availability of registered medicines [9, 11].

According to World Health Organization (WHO), Africa has 54 NRAs with varying capacities, but most cannot perform the critical regulatory functions [12]. A WHO report estimates that only 30% of NRAs among its Member States can effectively and efficiently regulate medical products in their countries [12]. WHO and its Member States require that medical products be registered; this involves the regulatory review of the quality, safety and efficacy data, good manufacturing practice inspection and licensing of the concerned manufacturing site before approval for marketing [10, 11, 13]. Such medical products may or may not have obtained the relevant authorization from the NRA in the source market [14, 15]. The 2017 WHO definition of poor-quality medicines includes “Unregistered/unlicensed medical products that have not undergone evaluation and/or approval by the National or Regional Regulatory Authority for the market in which they are marketed/distributed or used, subject to permitted conditions under national or regional regulation and legislation”[16]. There is evidence to demonstrate that unregistered medicines are associated with an increased incidence of adverse drug reactions and that despite advances in medicine regulation and guidance from professional organizations, the use of unregistered medicines [17, 18] in at-risk populations has not reduced [19, 20]. Moreover, the prevalence and impact of unregistered medicines is greater in low and middle-income countries due to the less mature regulatory systems as well as financial and human capacity constraints [21].

Ineffective delivery of quality healthcare services has led to shortages of medicines worldwide, including the Southern African Development Community (SADC) region [22–25]. Medicines shortages can be worsened in the absence of functional NRAs, exposing the public to potentially unsafe and spurious medical products. Medicines shortages can also be caused by changes in pharmaceutical manufacturers' marketing strategy, supply chain challenges, unexpected surges in demand, production-related issues, global pandemics such as Ebola and Covid-19, and natural disasters such as cyclones, among others [22, 23, 26].These factors create a vacuum for the availability of the medicines approved by the NRAs, leading to the demand and the use of medicines that have not undergone regulatory review approval process. Therefore, the importation and distribution of unregistered medicines is a global phenomenon that is often necessary to bridge the supply chain gaps, and this is expected to continue into the foreseeable future.

To objectively assess each NRA and create an opportunity for strengthening regulatory systems, WHO developed a Global Benchmarking Tool (GBT) [9]. The GBT assesses the strengths and weaknesses in the various regulatory functions and scores the system in terms of maturity levels (ML), ranging from 1 to 4 [2, 17]. In addition, the GBT assesses the availability of legal provisions, policies, manuals, guidelines, Standard Operating Procedures (SOPs), infrastructure, and availability of adequate and skilled personnel, which are all critical factors for effective regulation of medicines[2, 13, 17]. The use of unregistered medicines is expected to continue for the foreseeable future, raising concern globally, as documented in previous studies [27]. Of concern is that unregistered medicines' quality, efficacy, and safety remains unknown to the importing countries [28]. Therefore, to safeguard public health, the approval processes for importing and distributing unregistered medicines should be robust to mitigate the potential risk posed by such exemptions from the normal registration processes required to protect public health. The potential increase in the risk of adverse medical events or lack of therapeutic effect is also a cause for concern emanating from the importation of unregistered medicines [16, 27].

To our knowledge, the legal provisions and requirements set by each NRAs in the SADC region when approving the importation and distribution of unregistered medicines, similarities, differences, robustness, and challenges remain unknown as the processes are not usually published; hence no opportunity for knowledge sharing and or transfer. The objectives of this study were to review and determine the level of implementing the legal provisions and requirements for the importation of medicines across the SADC NRAs. The study provides baseline data for creating a regional guideline for good practices when importing unregistered medicines to protect public health. The identified gaps can be included in the institutional development plans for each NRA to enhance the regulatory systems strengthening within the region.

## Method

### Sample

SADC has 16 Member States. The legislative instruments and guidelines from 15 SADC countries, namely Angola, Botswana, Comoros, Democratic Republic of Congo (DRC), Eswatini, Lesotho, Madagascar, Malawi, Mozambique, Namibia, Seychelles, South Africa, Tanzania, Zambia, and Zimbabwe, were included in the analysis. No response or data was obtained from Mauritius.

### Source of data

The study reviewed the legislative provisions and the requirements for the approval of importation and distribution of unregistered medicines by 15 SADC NRAs, and two comparators Canada and UK, drawn from developed settings. Convenient selection of the two comparators was applied in this study. These two reference-NRAs are also listed as WHO-listed authorities, i.e., considered stringent regulatory authorities (SRAs) by WHO [29]. Data on the provisions and the requirements for the approval of importation and distribution of unregistered medicines were extracted from the legislation (laws and regulations) and guidelines of the NRAs by the researchers. Additionally, 15 independent reviewers drawn from each of the NRAs were tasked to review legislative provisions and the requirements for the approval of importation and distribution of unregistered medicines from their respective NRAs. A set of 18 parameters adopted from the WHO guideline on import procedures of medical products was used in the review process (table 1) [20].The themes were based on good regulatory practices for marketing authorization as detailed in WHO guidelines [30]. The parameters were measured on a 4-point scale based on the GBT: no formal approach (ML 1); reactive approach (ML 2); well-functioning and integrated regulatory systems (ML 3) and regulatory systems operating at an advanced level of performance and continuous improvement" (ML 4) [2, 19].

**Table 1:** Template of parameters for comparison of the content of the NRA legislation and guidelines^a^

Legislative Provisions or Enabling parameters
Legal provision allowing importation of unregistered medicines
Legal provision for allowing approval of donations
Guidelines
**Quality Parameters**
Requirement for GMP Approval (Inspection/Desk Reviews/ Reliance/ Recognition)
Requirement for Pre-Distribution Analysis
Requirement for Batch Specific Data such as Certificate of analysis
Requirement for Pre-Distribution Inspection
**Efficacy Parameter**
Requirement for Registration in the Country of origin or Recognized Jurisdictions
**Safety Parameters**
Requirement for Post Market Surveillance
Requirements Specific for Dosage Forms
Requirement for Product Specific Data (Stability, Pharmacovigilance Reports, Product Quality Reviews)
Requirement for product brochure containing chemical, pharmaceutical, pre-clinical pharmacological and toxicological data and where applicable, human, or animal pharmacological and clinical data with the medicine concerned
Requirement for Supply History
**Other Parameters**
Requirement for assessing if there is a registered alternative or the registered option has not been imported in the past 6 months
Requirement for a Dossier to have been submitted
Requirement for Availability in EML
Requirement for rationale why an unregistered medicine is required
Requirement for restriction to emergencies, disease outbreak, neglected disease and shortages

^a^These are ad hoc indicators that were developed for this research based on WHO GBT, WHO guideline on import procedures of medical products and requirements set out by SRAs

### Statistical analysis

The Relative Implementation Index (RII) is a derivative of the Relative Importance Index [31–33] was used to assess the level of implementation of the legislative provisions and the requirements for the approval of importation and distribution of unregistered medicines, overall, for the NRAs, across each parameter, and for each of the NRAs. The RII = ΣW/ (A*N), where W is the weighting given to each indicator (ranging from 1 to 4), A is the highest weight (i.e., 4 in this case), and N is the total number of indicators assessed [34]. All data analysis was carried in the STATA software package.

## Results

### Implementation of legislative provisions and guidelines

The level of implementation of the recommended standards is approximately 73% for importing unregistered medicines, 58% for receiving donations, and 54% for the use of standard operating procedures and guidelines.

### Implementation of the requirements for assessing the quality of imported medicines

Implementing the requirements for verifying that the manufacturing site has Good Manufacturing Practice (GMP) approval was estimated to be 53%, 23% for carrying out pre-distribution analysis, 36% for requesting and verifying batch specific data such as certificate of analysis, and 33% for pre-distribution inspection of received consignments across the NRAs.

### Implementation of the requirements for assessing the efficacy and safety of imported medicines

The level of verification for registration in the country of origin or recognized jurisdictions was estimated to be 33% across the NRAs, 54% for implementing post-market surveillance activities and 14% had implemented the special requirements for specific dosage forms and product-specific data. The level of implementing the requirement to request and review product brochures containing chemical, pharmaceutical, pre-clinical pharmacological and toxicological data and, where applicable, human, or animal pharmacological and clinical data with the medicine concerned was estimated to be 28% across the NRAs. Similarly, a 24% implementation level was noted across NRAs for reviewing supply history.

### Implementation of other additional requirements

Assessing if a registered alternative or the registered option has not been imported in the past six months occurs in 48% of the NRAs, and 16% require a dossier to be submitted or commitment as part of approval process for importation. Only 12% implementation level was observed on the requirement for only importing medicines in the Essential Medicines List (EML), and 27% had implemented the requirement for applicants to provide justification for importing an unregistered medicine. The level of implementing the requirement for restriction to use unregistered medicines in emergencies, disease outbreaks, neglected disease and shortages was estimated to be 22% across the NRAs.

### Implementation level by country

**Fig. 1:**
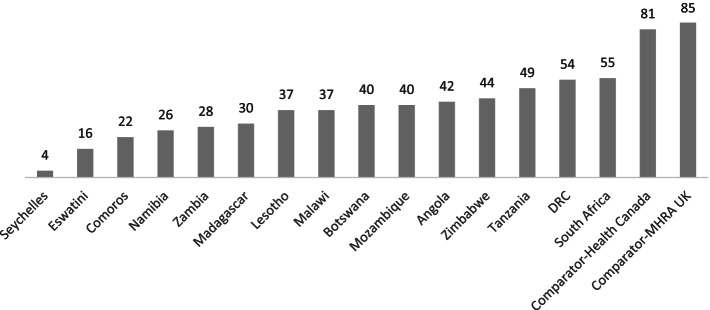
Relative Implementation Index (%) by country including examples of comparators from developed settings

The overall level of implementation of the legal provisions for the importation of medicines across all the NRAs in SADC was estimated to be approximately 44%, ranging from 4% to 55%, while that for the comparators Canada and the United Kingdom was estimated to be approximately 81% and 85%, respectively (Fig. 1).

## Discussion

To our knowledge, this is the first study to explore the regulations and requirements for the importation of unregistered medicines and receiving donations in the SADC region. The RII was used as a measure of the level of implementation for regulation of the importation of medicine. It is important to note that a single RII alone may not be useful as a reference for measuring adequacy, but useful in evaluating the differences in the levels of implementation by themes and or overall themes of the countries. Furthermore, in this study, we analysed review responses from the principal investigator combined with that of a reviewer from the country’s respective NRA, hence the few numbers of responses available for this study could not support by theme analysis, thus the reason the study concentrated more on the per country analysis. The analysis of the RII by theme and then overall by country needs to be considered for studies that obtain a considerable number of various reviewers’ responses. Our results are not correlated with the maturity level status of the NRA because there is insufficient data available on the maturity level of countries included in the study. Only one country, Tanzania, was assessed and the results are publicly available. It would be worth investigating the correlation of the results in the GBT relating to the handling of unregistered medicines and specific indicators used in our research if more of the NRA assessment data are publicly available. WHO is finalizing the performance evaluation as part of future NRA assessments. Further research with GBT plus performance evaluation may be needed to verify the correlation of RII and maturity levels.

Thirteen out of the 15 SADC NRAs were estimated to be below 50% implementation level compared to above 80% for the two reference NRAs. All the SADC NRAs had a score of less than 60%. The wide-ranging implementation index scores also confirm the varying regulatory capacities within the region. Our results show that the SADC countries have not fully implemented most of the expected risk-based requirements for approving requests for importing unregistered medicines. The review conducted in this study shows that the SADC countries have lower levels with regards to implementation of WHO set legislation and guidelines regarding to import procedures for medical products compared to comparators drawn from developed set-ups on import procedures for medical products [18, 35].

Our study focused on the regulation of unregistered medicines, an area often neglected and not fully addressed in ongoing efforts to strengthen regulatory systems. Nonetheless, the results are consistent with previous reports; for example, the estimates that only 30% of NRAs among SADC Member States can effectively and efficiently regulate medical products in their countries [36].The results are also consistent with other studies that observed that legal frameworks lacked or were fragmented in most African countries [2, 6] Additionally, some studies have shown that implementing medicines control regulation on the importation, use, and reporting of adverse drug reactions in developing countries is by far still too low compared to the levels in high-income countries [37, 38]. This may result in NRAs in SADC, or similar settings, allowing the importation of medicines whose quality is unknown and not being adequately assessed.

SADC is a region with diverse countries across several dimensions, ranging population sizes, geographical sizes, the size of the pharmaceutical market, economic levels, and regulatory capacities. The study, therefore, encompassed many different factors, and results can be generalized to countries in Africa or similar settings. The GBT is considered a well-established tool through a robust process and applied globally. Therefore, the indicators used in the study are considered validated. The basis for requirements used in the study was the requirements in WHO guidelines on import procedures of medical products, requirements for obtaining marketing authorization, and those set out by ML4 NRAs.

Given that unregistered medicines should be imported under exceptional circumstances and in some cases during emergencies, it may not always be feasible to implement all the requirements for granting marketing authorizations. Nonetheless, even in those circumstances, there remains a need to assess the medicines' quality, efficacy, and safety to protect public health based on a risk-based approach. Therefore, the results are based on comprehensive parameters that were considered sufficient to reflect the implementation of the regulatory function for exemptions.

Analysis of legislation and guidelines is robust and objective; however, interpretation of the results should contextualize the different legislative systems across the countries and implications at the operational level when implementing the legislation provision in practice. Therefore, further studies analysing data on the importation of unregistered medicines and practices among the regulators are required. In other words, the implementation index values alone may not directly indicate public health risk for individual countries. Other factors should be considered, for example, the extent to which unregistered medicines are supplied in the public sector, the supply chain of such unregistered products, and the quality assurance mechanisms implemented by the procurement agencies. For instance, United Nations Agencies and other international organizations largely procure WHO-prequalified products or products approved by SRAs. Therefore, while these products may be unregistered in the SADC NRAs, their quality, safety, and efficacy are known and established [28, 29].

## Conclusion

Implementing the standard guidelines for importing unregistered medicines in SADC is still very low, with an overall implementing index of 44% compared to the reference NRAs with index values above 80%. The region is heterogeneous, with a wide range among the NRAs. The low implementation levels of recommended standards for unregistered medicines increase the risk of potentially exposing the population in the region to unsafe medical products of variable quality and effectiveness. Inconclusion, the SADC countries should harmonise and share experiences to implement reliance models when regulating unregistered medicine. However, the impact of harmonisation on this regulatory function will need to be investigated in future studies.

## Data Availability

The study is yet to publish the data to the public. However, the data can be available upon request and approval from the corresponding author.
